# Research progress on the application of optical coherence tomography in the field of oncology

**DOI:** 10.3389/fonc.2022.953934

**Published:** 2022-07-25

**Authors:** Linhai Yang, Yulun Chen, Shuting Ling, Jing Wang, Guangxing Wang, Bei Zhang, Hengyu Zhao, Qingliang Zhao, Jingsong Mao

**Affiliations:** ^1^ State Key Laboratory of Molecular Vaccinology and Molecular Diagnostics, Center for Molecular Imaging and Translational Medicine, Department of Laboratory Medicine, School of Public Health, Shenzhen Research Institute of Xiamen University, Xiamen University, Xiamen, China; ^2^ School of Medicine, Xiamen University, Xiamen, China; ^3^ Department of Imaging, School of Medicine, Xiamen Cardiovascular Hospital of Xiamen University, Xiamen University, Xiamen, China; ^4^ Department of Radiology, Xiamen Key Laboratory of Endocrine-Related Cancer Precision Medicine, Xiang’an Hospital of Xiamen University, Xiamen, China

**Keywords:** optical coherence tomography, cancer imaging, oncological diseases, imaging technique, tumor diagnoses

## Abstract

Optical coherence tomography (OCT) is a non-invasive imaging technique which has become the “gold standard” for diagnosis in the field of ophthalmology. However, in contrast to the eye, nontransparent tissues exhibit a high degree of optical scattering and absorption, resulting in a limited OCT imaging depth. And the progress made in the past decade in OCT technology have made it possible to image nontransparent tissues with high spatial resolution at large (up to 2mm) imaging depth. On the one hand, OCT can be used in a rapid, noninvasive way to detect diseased tissues, organs, blood vessels or glands. On the other hand, it can also identify the optical characteristics of suspicious parts in the early stage of the disease, which is of great significance for the early diagnosis of tumor diseases. Furthermore, OCT imaging has been explored for imaging tumor cells and their dynamics, and for the monitoring of tumor responses to treatments. This review summarizes the recent advances in the OCT area, which application in oncological diagnosis and treatment in different types: (1) superficial tumors:OCT could detect microscopic information on the skin’s surface at high resolution and has been demonstrated to help diagnose common skin cancers; (2) gastrointestinal tumors: OCT can be integrated into small probes and catheters to image the structure of the stomach wall, enabling the diagnosis and differentiation of gastrointestinal tumors and inflammation; (3) deep tumors: with the rapid development of OCT imaging technology, it has shown great potential in the diagnosis of deep tumors such in brain tumors, breast cancer, bladder cancer, and lung cancer.

## Introduction

OCT is a noninvasive optical imaging technique, that can capture high-resolution and three-dimensional (3D) images on biological tissues with label-free. Huang et al., in 1991 (1), first proposed a concept of OCT. Through the weak coherent light interferometer theory, images of biological tissues with excellent axial resolution (<10 μm) can be obtained in real-time utilizing near-infrared (NIR) light waves reflected by microstructures within the tissue (2). Based on the above advantages, OCT is widely used in a variety of biomedical fields, including ophthalmology, dentistry, dermatology, oncological and cardiovascular, among others.

In biomedical fields, optical imaging technology is extensively used such as Laser scanning confocal imaging (LSCI), Two-Photon imaging, Fluorescence imaging (FI), Laser speckle imaging (LSI), Laser doppler imaging (LDI) and OCT, etc. Although LSCI and two-photon imaging techniques could provide high spatial resolution images of biological tissues, imaging under aqueous or oily objectives requires contact with tissues, while the relatively small imaging view field and low penetration depth make them impractical for clinical application (3, 4). In terms of LSI, it is also difficult to perform depth-resolved *in vivo* 3D imaging due to depth limitation, even though it can offer high-resolution, non-contact imaging (5). A millimeter- resolution LDI can only monitor microcirculatory vessels (6). FI involves the use of fluorescent materials as imaging labels (7), which may cause adverse effects like allergies. In contract, OCT offers a non-invasive method for imaging tumor tissue at multiple scales, with high contrast and resolution *in vivo*, as well as displaying high endogenous contrast in biological tissues (8). Moreover, OCT allows for deep penetration of tissue up to 2 mm and can rapidly produce 3D images with high temporal resolution (9, 10).

**Figure 1:**
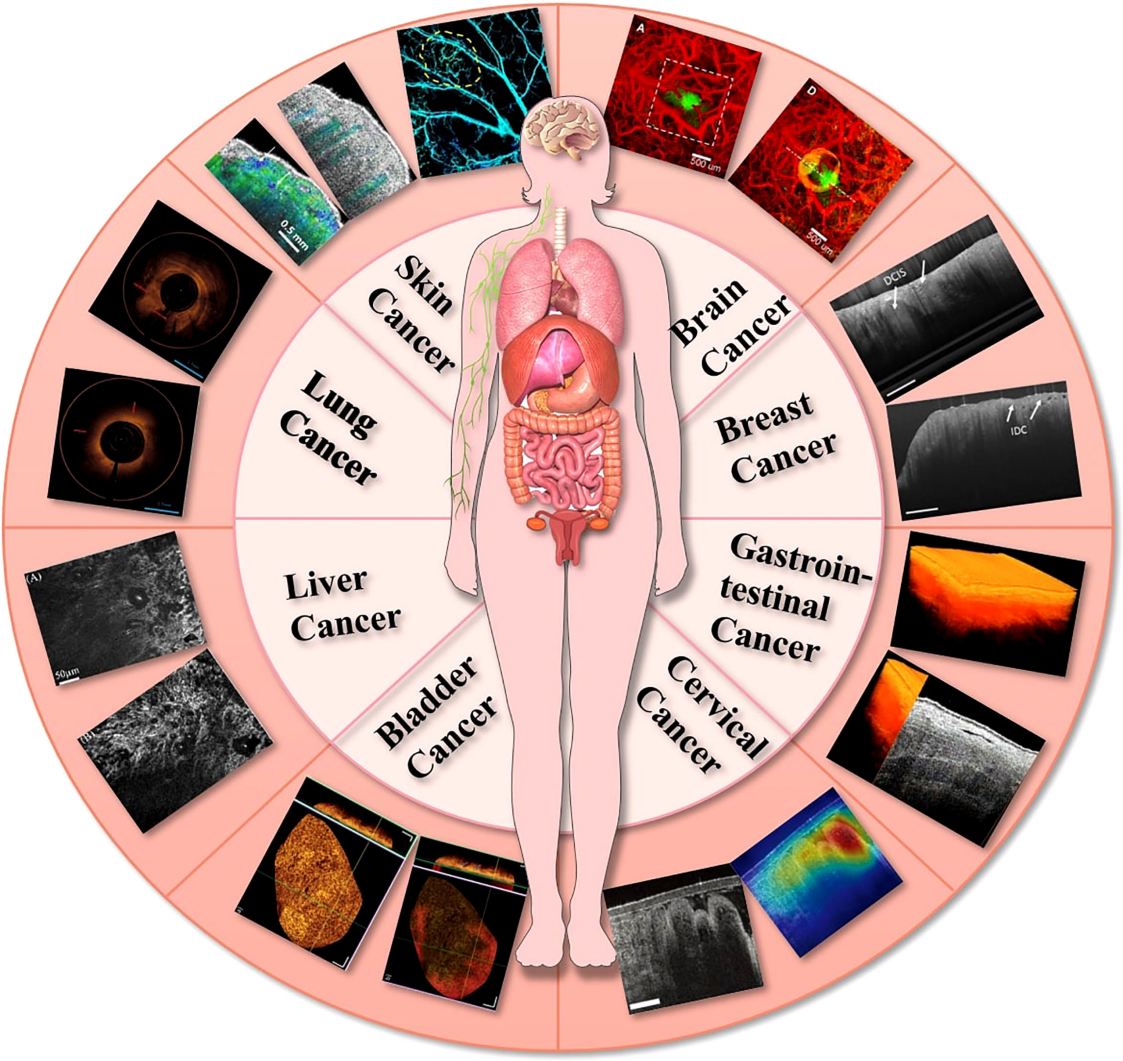
Application of OCT in the field of oncology.

After the development of OCT, it was widely used in ocular imaging for glaucoma (11–13), macular degeneration (14–16), retinal vein obstruction (17–19), diabetic retinal microaneurysm (20, 21), uveitis (22–24), etc. The advances in OCT imaging applications for ophthalmology were detailed in relevant reviews in 2018 (25), 2019 (22), 2020 (26), and 2022 (27). OCT has also contributed to the fields other than ophthalmology due to its miniaturization and integration with catheters and endoscopes (**Figure 1**). Hence, an overview of OCT imaging technology will be given in this article, along with its recent developments in oncological diseases: (1) superficial tumors: OCT could detect microscopic information on the skin’s surface at high resolution and has been demonstrated to help diagnose common skin cancers; (2) gastrointestinal tumors: OCT can be integrated into small probes and catheters to image the structure of the stomach wall, enabling the diagnosis and differentiation of gastrointestinal tumors and inflammation; (3) deep tumors: with the rapid development of OCT imaging technology, it has shown great potential in the diagnosis of deep tumors such in brain tumors, breast cancer, bladder cancer and lung cancer. In addition, the possible future development direction of OCT is prospected.

## Development of the OCT

Low coherence interference of light is the basis for OCT, which is similar to ultrasound imaging in principle. Based on the Michelson interferometer, the OCT imaging equipment architecture obtains tissue reflection signals with depth resolution characteristics by detecting an interference signal formed between the reflected light of the reference lens and the backscattered light of the sample objective. By changing the relative position of the reference mirror, the intensity of the backscattered light of tissue can be detected at different depths. In the axial direction, echo sequences with different depths form an intensity distribution (A-scan). Multi-points A-Scan reconstruction results in a two-dimensional (2D) cross-sectional image of the tissue, called B-Scan. And then the 3D structure of the tissue can be obtained by reconstructing the B-Scan at different locations (28, 29).

**Figure 2:**
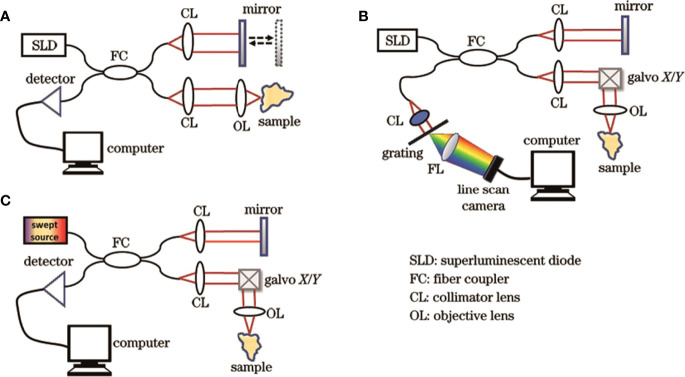
Structural diagrams of three generations of OCT systems. **(A)** TD-OCT; **(B)** SD-OCT; **(C)** SS-OCT (30). Copyright 2022, www.opticsjournal.net.

OCT imaging technology has gone through three generations, namely time-domain OCT (TD-OCT), spectral-domain OCT (SD-OCT), and swept OCT (SS-OCT), with the advancement of laser and computer technology and the optimization of imaging algorithms (**Figure 2**). The first-generation OCT system based on time-domain detection relied on time delay measurement of the reflected signal from tissue relative to the reflected signal from the reference mirror. The optical signal reflected from the target tissue superimposes and interferes with the optical signal reflected by the reference mirror, resulting in the formation of the OCT. This procedure requires mechanically shifting the reference mirror, thereby changing the depth of the tissue being scanned (31, 32). However, with the advancement of technology and technology and for different needs, TD-OCT has emerged many variants, such as line-field confocal OCT (LC-OCT) (33, 34), full-field OCT (FF-OCT) (35), polarization-sensitive OCT (PS-OCT) (36), etc., to achieve more efficient and wide applications in the clinic.

Unlike TD-OCT, the reference mirror of the reference arm is fixed in the second-generation SD-OCT structure. The interference of the optical signal can be achieved by varying the frequency of the light source, and the data acquisition rate can be raised by 45-100 times (37). Simultaneously, SD-OCT can measure the spatial and structural information on all echo delays (axial pixels) by evaluating the interference spectrum between the light signal from the rest-reference mirror and the light signal reflected from biological tissue (38).

Though SD-OCT and SS-OCT use Fourier domain techniques, spectrometers and high-speed line scan cameras are utilized to measure the interferometer spectra of the SD-OCT. On the other hand, SS-OCT detects OCT signals with sweep light sources and photodetectors. Moreover, the SS-OCT has a high-speed scan rate and a tunable scanning laser, resulting longer wavelengths than traditional spectral-domain OCT (20, 39). Therefore, the third-generation SS-OCT has faster scan speeds, higher scan densities, less deeply dependent signal-to-noise ratios, and higher resolutions. These properties enable them to reduce the impact of motion artifacts and better visualize tissues such as blood vessels while imaging larger areas, improving the quality of OCT *in vivo* imaging, and expanding its usage in biomedical research. Toward Pi Company has recently developed an SS-OCT system with 400,000 cycles per second that can rapidly reach an imaging depth of 6 mm. Simultaneously, the scanning length of a single image reaches 24 mm, and the axial resolution reaches as high as 3.8 μm. The images shine in the commercial field of ophthalmic OCT because of its excellent imaging parameters in both local and foreign markets (40).

Optical coherence tomography angiography (OCTA) is a kind of vascular imaging technology, belonging to the SS-OCT, which can visualize and quantify the morphological information of blood vessels by detecting the red blood cell (RBC) movement of the intravascular dynamic scattering signal (41). It has become the “gold standard” in the field of ophthalmic diagnostics. Currently, applications in the field of oncology are also widely studied.

## Application of OCT in oncology

Cancer is a leading cause of death worldwide and most patients are in the middle and advanced stages of treatment due to the subtle onset of early cancer and the inconspicuous symptoms. Traditional medical imaging methods focus on morphological tumor diagnosis, however, association of the imaging characteristics with early cancer is not apparent. Therefore, achieving multi-angle, all-round imaging and diagnosis of early cancer occurrence and progression from structural and functional levels, and providing timely radical treatment, is a significant component in the long-term survival of cancer patients. OCT has become a novel approach to early cancer diagnosis due to its rapid development.

### Application of OCT in superficial tumors

The skin is not only the largest and most accessible organ of the human body, but it also has relatively clear layered structures. Therefore, the microstructural information of the skin surface can be easily visualized using the OCT of near-infrared light. OCT not only generates micron-level images of living skin with a depth of 2 mm, but also is convenient, real-time, dynamic, great repeatable, and inexpensive. Hence, it is widely used in the diagnosis of superficial tumors. It was first used in 1997 to diagnose skin lesions as an additional tool for diagnosing and monitoring skin lesions (42). The high-resolution OCT detects the epidermis, dermis, appendages, and blood vessels of the skin, as well as evaluates the response to treatment of some diseases. OCT has already been demonstrated to help diagnose common skin cancers.

OCT is considered to be an advantageous diagnostic method for non-melanoma skin cancer, offering potential for diagnosis in the early stages of the disease. Non-melanoma skin cancers are generally classified as basal cell carcinoma (BCC) and squamous cell carcinoma (SCC) (43).

In 2021, an international consensus statement on Basal cell carcinoma (BCC) OCT, including BCC term sets for different subtypes was proposed. The publication of this statement helps implement OCT imaging of basal cell carcinoma in clinical and research settings (44). Adan et al. used the established diagnostic value of OCT features in 99 patients to determine whether OCT features could accurately distinguish BCC from non-BCC and BCC subtypes. The results showed that a limited number of OCT features were able to distinguish superficial BCC from non-superficial BCC and non-BCC lesions. The diagnostic method was able to detect 97.8% of BCC lesions, 84.3% of superficial BCC lesions and 98.8% of non-superficial BCC lesions (45). The LC-OCT technique, which combines reflex confocal microscopy with OCT technology, explains the basal cell carcinoma characteristics under LC-OCT examination and offers a theoretical basis for the diagnosis, classification, and treatment of later basal cell carcinoma (33, 34).

Cutaneous squamous cell carcinoma (SCC) is another common non-melanoma skin cancer that, unlike BCC, has the potential to metastasize. Early recognition and treatment are critical to reducing this risk, and actinic keratosis (AK) is considered a precursor lesion in SCC (46). Zhou et al., used an SD-OCT to image AK lesions of varying degrees in mice, which showed that the irregular wavy dermal-epidermal junction (DEJ) and persistent thickening of the epidermis are useful diagnostic parameters for AK. It demonstrates the great potential of OCT for non-invasive diagnosis of precancerous lesions (47). Cinotti et al. imaged 158 patients preoperatively using LC-OCT devices and performed histological examinations postoperatively. Conclusions show that LC-OCT is a new non-invasive imaging technique that can identify the main features of AK and SCC, which can help clinicians detect cellular and structural changes in keratinocyte skin tumors in real-time (48). Ho et al. based on a convolutional neural network (CNN) developed a mouse skin SCC classification model that integrates a FF-OCT device. This model provides a rapid, non-invasive, and accurate SCC classification, achieving 87.12% and 90.10% classification accuracy at the image level and tomography image level, respectively (35).

Conventional OCT is considered to be less sensitive for detecting early-stage melanoma, but it has the highest sensitivity compared to other techniques such as confocal microscopy, ultrasonography, and multispectral imaging. The imaging results of high-definition OCT (HD-OCT) and speckle variance OCT (SV-OCT) for melanoma are more positive than conventional OCT. However, due to the limited data available, more reports are needed to draw conclusions about their effects (49).

**Figure 3:**
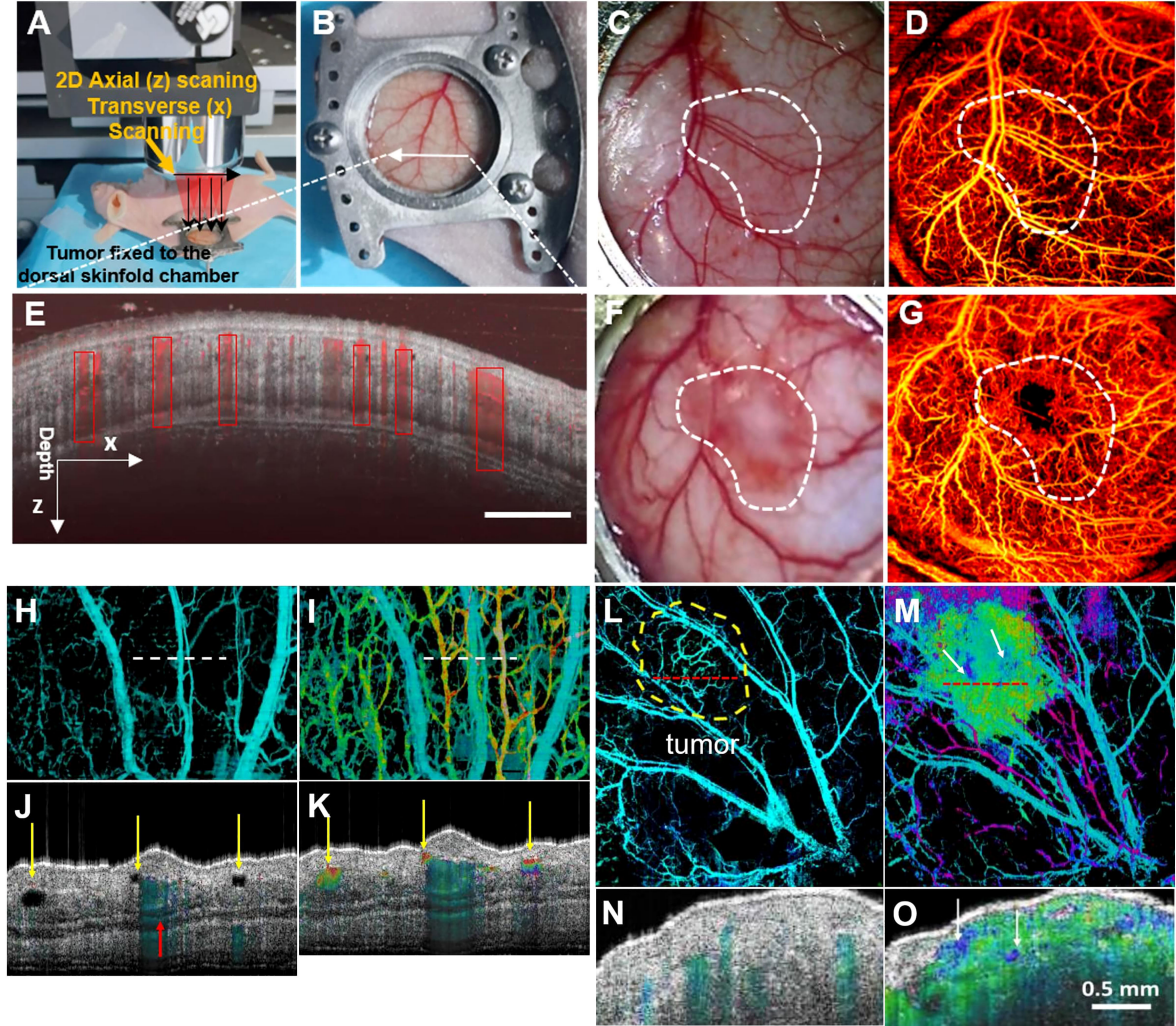
**(A)** Schematic of subcutaneous tumor-bearing nude mice dorsal window imaging; **(B)** Subcutaneous tumors of nude mice with tumors enlarge the skin window chamber images; **(C)** The zoom-in skin window chamber image in the healthy nude mice; **(D)** The corresponding enface microvascular image *in vivo* is shown in **(C)**; **(E)** Representative tissue cross sectional structural image (gray) and blood flow image (red border); **(F)** The zoom-in skin window chamber image in the subcutaneous tumor-bearing nude mice; **(G)** The corresponding enface microvascular image *in vivo* is shown in **(F)** (50); Copyright 2021, Wiley. **(H)** Normal vascular OCT images; **(I)** OCT images of angiogram and lymphangiography. The dotted line indicates the position of the cross-section image in A-B. **(J, K)** normal angiography and lymphangiography OCT en-face images. The red arrow indicates a large blood vessel, and the yellow arrow indicates the lymphatic vessel. **(L)** Melanoma vascular OCT image. **(M)** OCT images of melanoma angiography and lymphangiography. The dotted line indicates the position of the cross-section image in **(E–F)**. **(N, O)** Melanoma angiography and lymphangiography OCT en-face image (52). Copyright 2020, American chemistry society.

**Figure 4:**
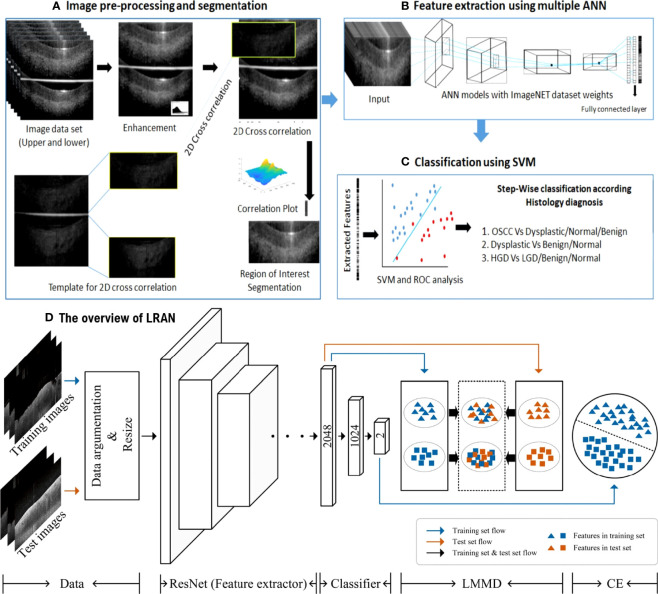
**(A-C)** ANN-SVM based image analysis pipeline (53); Copyright 2021, MDPI. **(D)** The overview of LRAN (58) Copyright 2022, Springer.

OCT technology offer changes in tumor microvascular before and after treatment to assess tumor microvascular response to nano therapy. This creates the theoretical and technical base for developing new tumor-specific diagnostic and treatment approaches (50). Welzel et al. observed blood vessels in skin and malignant melanoma using Dynamic optical coherence tomography (D-OCT) D-OCT and proposed that increased blood vessel density and irregular vascular patterns were more common in melanoma and more common in higher-stage melanoma (51). OCT can visually exhibit microscopic characteristics within tissues and distinguish lymph node tissue and surrounding adipose tissue, revealing changes in nodular microstructure during metastatic tumor invasion (**Figure 3**). Si et al. generated “flow-gated” spectral OCT images using a dual-band signal processing algorithm that demonstrates lymphatic drainage pathways for melanoma blood vessels and peritumoral tissue at micron-scale resolution (**Figure 4**) (52). **Table 1** summarizes the imaging capabilities of OCT in superficial tumors of the skin.

**Table 1:** The imaging capabilities of OCT in superficial tumors.

Cancer	Authors	Main Findings
Cutaneous melanoma	Von Knorring et al. (2022) (54); Schuh et al. (2022) (55)	OCT can distinguish benign and malignant pigmented cutaneous tumors
Cutaneous basal cell carcinoma	Gust et al. (2022) (56); Suppa M et al. (2021) (33); Verzì et al. (2021) (57)	LC-OCT can describe the characteristics of basal cell carcinoma at the bedside for differential diagnosis of basal cell carcinoma and for typing of basal cell carcinoma and assessment of response to treatment of BCC.
Squamous cell carcinoma of the skin	Ho et al. (2021) (35)	FF-OCT can provide fast, non-invasive, and accurate SCC classification with high accuracy.
Oral cancer	Yuan et al. (2022) (58); Trebing et al. (2021) (59); Ilhan et al. (2020) (60); Chen et al. (2020) (61)	OCT can assess structural changes in oral epithelial cells, distinguish oral cancer from precancerous lesion tissue, and conduct noninvasive screening, detection, evaluation of differentiation, and staging oral dysplasia and early cancer.

The introduction of non-invasive, efficient, and cost-effective screening tools will enhance the early detection of oral cancer and hence, the patient’s lifespan. A Local Residual Adaptation Network (LRAN) model based on deep learning technology was developed for qualitative and quantitative analysis of oral cancer OCT image datasets with high accuracy and sensitivity (58). Furthermore, a 3D technique of SD-OCT was developed for evaluating the structural changes in oral epithelial cells, which improved the time efficiency and quality of diagnosing epithelial lesions (59). Automatic image processing algorithms in OCT images can differentiate between heterotypic oral potentially malignant lesions (OPML) and malignant lesions, resulting in high sensitivity. Evidence is provided by using reliable and low-cost OCT instruments as point-of-care devices in resource-constrained settings and potential clinical applications in oral cancer screening and surveillance (53).

### Application of OCT in gastrointestinal tumors

OCT has shown significant potential in cavity organ tumors using techniques, such as endoscopy, catheterization, and laparoscopy. OCT imaging can distinguish between the four layers of the stomach wall structure, namely the glandular epithelium, mucosal muscle layer, submucosal layer, and muscle layer, where the submucosal layer is visible to the blood vessels. Jansen et al. used prospective research to investigate 26 patients with esophageal cancer. Calculate the contrast of plaques in an M-mode scan to distinguish between blood flow areas and resting tissues. This study is the first to confirm the OCT imaging of gastric tissue and blood flow detection *in vivo* during surgery in esophageal cancer patients, reducing the occurrence of anastomotic leakage after operation and improving surgical outcomes for patients (62).

**Figure 5:**
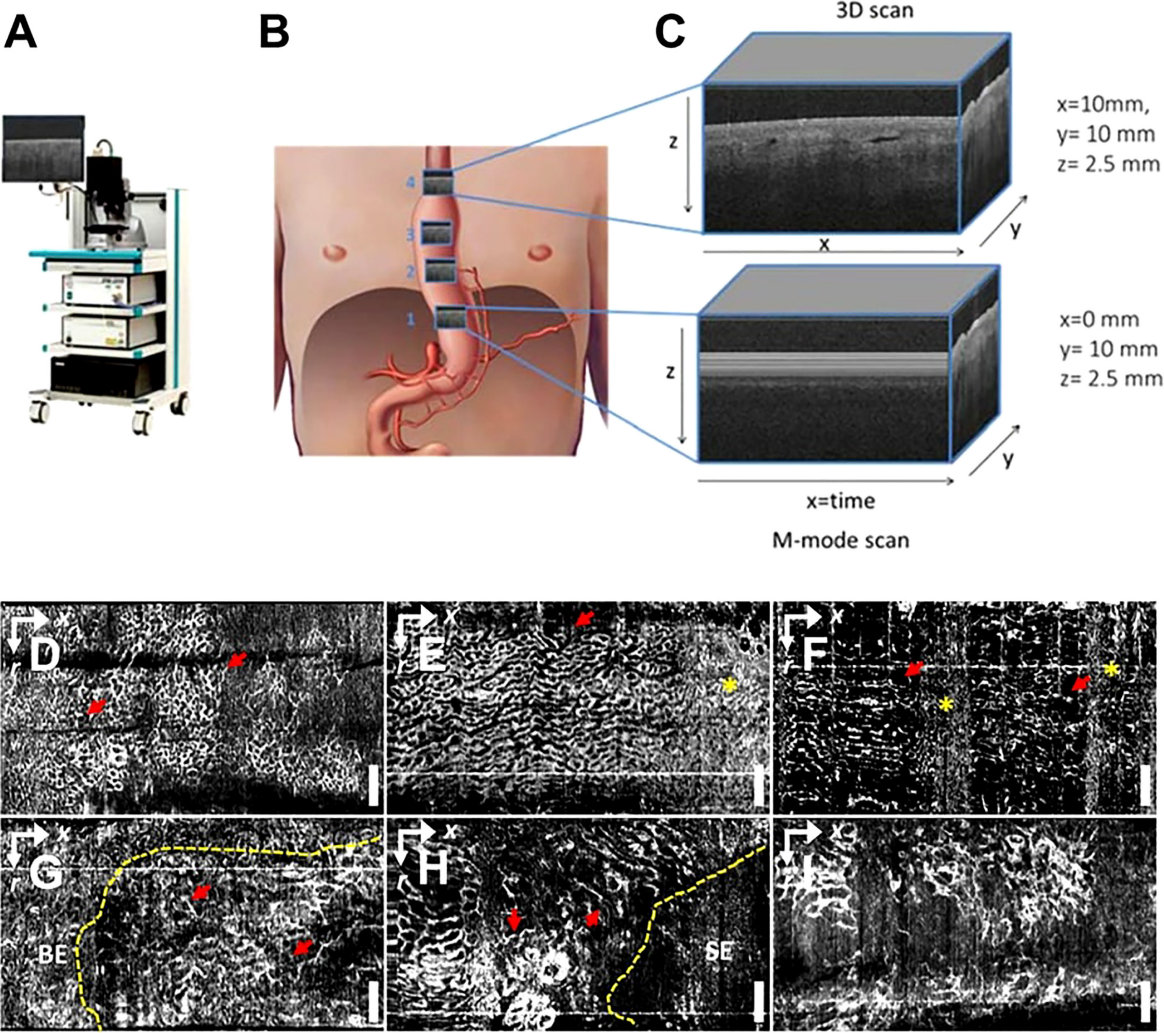
**(A)** Schematic diagram of a commercial OCT; **(B)** Gastric tube perfusion areas and **(C)** ROI region OCT grayscale image, cross-sectional OCT image showing vessels shadow (62); Copyright 2018, MDPI. OCTA vascular imaging of **(D-F)** non-dysplasia and **(G-I)** dysplasia BE (63). Nanoparticles targeting the hypoxic tumor microenvironment. Copyright 2017, ELSEVIER.

OCT imaging of the esophageal and gastrointestinal parietal structures can detect various digestive tract diseases because tumors and normal tissues exhibit different light scattering patterns on OCT images. Lee et al. used volumetric OCTA imaging and corresponding histological diagnosis of 52 dysplasia patients who received Barrett’s esophagus (BE) monitoring or endoscopic eradication of dysplasia, which can differentiate between low-grade dysplasia, low-grade dysplasia (LGD), and high-grade dysplasia (HGD) with the ability to visualize LGD/HGD-associated microvascular features with high accuracy (63) (**Figure 5**). Rodriguez et al. reviewed 14 studies, including endoluminal laser microscopy and OCT imaging of Barrett’s esophagus, and found that endoscopic imaging of Barrett’s esophagus with OCT and laser intraluminal microscopy could perform targeted biopsies and improve the probability of early detection of esophageal tumors (64).

OCT’s ability to image the layers of the gastrointestinal wall can be used to diagnose cross-wall inflammation in Crohn’s disease (CD) and differentiate it from ulcerative colitis (UC). Shen et al. used colonoscopic OCT to express the lamellar structures of colon wall disintegration. They discovered that the destruction of colon wall layers on OCT is a reliable indicator of CD transmural inflammation (65).

OCT has a critical role in achieving qualitative real-time analysis and targeted biopsy. Ding et al. used OCT images to differentiate dysplasia and cancer from normal colonic tissue (66). For the first time, Hariri et al. used endoscopic OCT and laser-induced fluorescence (LIF) OCT-LIF to demonstrate repetitive, minimally invasive, cross-sectional colon imaging in mice, observing the development of adenoma with atypical hyperplasia of the colonic mucosal epithelium, mucosal thickening. The mucosal/submucosal barrier can be uplifted and disrupted by mucosal cancer tissue (67). Harpel et al. used OCT to track the onset and development of colorectal cancer in mice. They discovered that OCT could be used to allow for the monitoring of morphological changes in the distal colon due to tumor development and the presence of lymphoid aggregates. In addition, the role of inflammation on tumor development and the immune system can be elucidated. So, they could be used as novel therapeutic agents to prevent disease progression and increase the efficacy of anti-cancer agents. OCT can also be useful for initiating early therapy and assessing the benefit of combination therapy targeting inflammation (68).

Overall, OCT imaging is useful in the early differential detection of gastrointestinal tumors. The intraluminal optical tomography scanner (62, 69)could become a helpful reference for rapid, low-cost, non-invasive light biopsy, early differential diagnosis, and treatment of gastrointestinal cancers (**Table 2**).

**Table 2:** The imaging capabilities of OCT in gastrointestinal tumors.

Cancer	Authors	Main Findings
Esophageal adenocarcinoma	Rodriguez et al. (2019) (64); Lee et al. (2017) (63)	OCTA can distinguish between LGD and HGD while showing LGD/HGD-related microvascular features with high accuracy. Endoscopic imaging using OCT and laser intraluminal microscopy allows for targeted biopsy to improve the probability of early detection of esophageal tumors.
Colon cancer	Kendall et al. (2022) (70)	OCT can show the “texture” of tissue well and is an excellent way to assess the mucosal thickness and the number of layers for quick identification and classification of tissues.
Gastric cancer	Jansen et al. (2018) (62)	OCT can perform real-time visual blood flow detection imaging in a surgical setting to evaluate the efficacy of surgery or drugs by using Vivo microcirculatory perfusion destined data.

### Application of OCT in deep tumors

Many research groups (71) have developed new OCT technologies to perform extensive studies in deep tumors with the rapid growth of lasers and computers.

**Figure 6:**
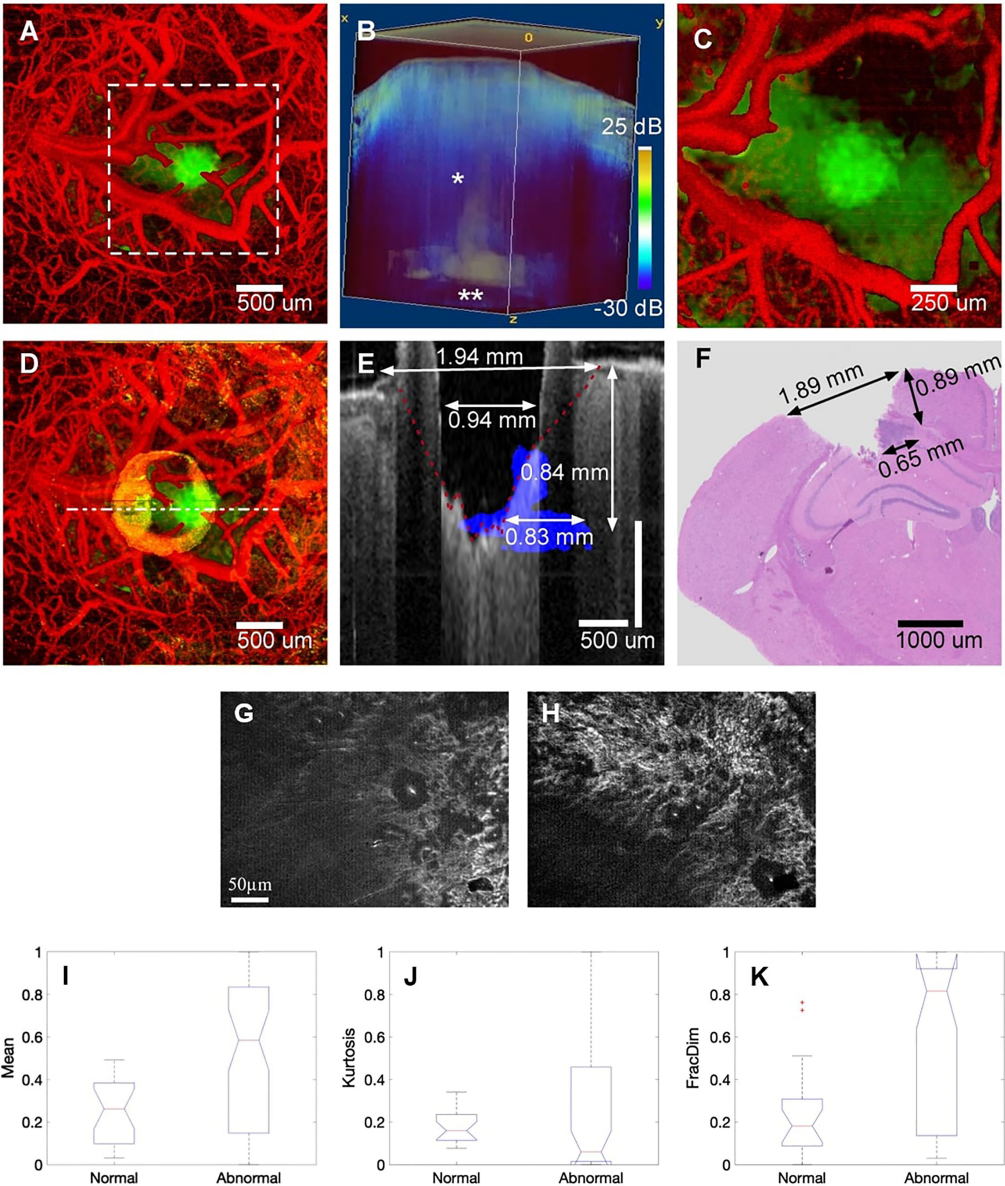
**(A)** Pretreatment of cerebral surface vascular construction (red) and tumor area (green) in mice. **(B)** Renders an image in 3D with an attenuation rate threshold mask superimposed on the OCT intensity (blue). **(C)** Maximum intensity projection after coagulation. **(D)** Maximum intensity projection after ablation. **(E)** After overlapping the tumor margins (blue) before ablation, stain the corresponding area with the post-ablation b-scan (gray) **(F)** H&E stained of the corresponding region (72); Copyright 2019, Theranostics. **(G)** FF-OCT image of normal and **(H)** cancerous hepatic cell. **(I-K)** Boxplot of selected features for the Mean, Kurtosis, and FracDim. mean and kurtosis are not sensitive in distinguishing between normal and cancerous hepatocytes, the mean and kurtosis are not sensitive in distinguishing between normal and cancerous hepatocytes, when the liver becomes cancerous, the value of the fractal parameter increases (88). Copyright 2020, Wiley.

OCT enables fast, wide-field, and label-free imaging of the living brain. In 2019, Katta et al. (72) used OCT to coagulate blood vessels and performed laser ablation of brain tumors (**Figure 6**). Yecies et al. published a new *in vivo* imaging approach using speckle-modulating OCT (SM-OCT) for label-free *in vivo* nerve and tumor edge identification in the same year. SM-OCT was used to show the white matter bundle and cortical layer structure in the brains of live mice. They identified the edges of glioblast tumors *in situ* in a mouse brain at an imaging of 10 μm (10).

Rapid and accurate evaluation of the intraoperative margin is vital for minimizing the resection rate in breast cancer. Using OCT images, Singla et al. used an active reverse-learning pre-trained inception-v3 CNN to distinguish between healthy and cancerous breast tissue. The method is highly sensitive, specific, and accurate (73). Likewise, Mojahed concluded that using CNN-based algorithms, it is possible to accurately identify malignant spots in OCT images (74).

Kansal et al. also developed a high-resolution automated full-field polarization-sensitive optical coherence tomography (FF-PS-OCT) system that was utilized to visualize 12 breast tissue samples, including four healthy tissues and eight malignant (cancerous) tissues. They used 106 OCT pictures to extract various phase features. This system can detect breast cancer models with up to 90.90% sensitivity and 85.0% specificity (75). Yang et al. analyzed the resection of normal breast tissue, breast cancer tissue, benign breast lesions, and axillary lymph nodes using FF-OCT and dynamic cell imaging (DCI). The findings reveal that FF-OCT and DCI have high accuracy in diagnosing breast cancer and have good diagnostic potential in breast surgery (76). Many research organizations have developed deep learning technology to improve qualitative leaps in image recognition and diagnostic characterization. More recently, Chen et al. created a computer-aided diagnosis (CADx) method that designs a contrastive texture learning strategy, with a sensitivity of 91.17% ± 4.99% for OCT image plaques. The specificity was 93.96% ± 4.72%, providing better interpretability based on texture features, which could lead to immediate clinical treatment (77).

Bladder cancer patients may benefit from OCT in addition to endoscopy for staging and grading. A prospective multicenter phase II trial revealed that OCT-assisted cystoscopy is a real-time, noninvasive, and maneuverable facility that increases the accuracy of bladder cancer staging and tumor invasion prediction (78). Xu et al. used intracellular motion (IM) as a dynamic contrast agent to track the distribution of urinary celiac cells. This contrast could provide a novel mechanism for OCT to accurately depict urothelial cancer cells’ the depth and kind of invasion to stage bladder cancer (79). Wurster et al. offered a piezoelectric fiber-optic scanner-based forward-imaging endoscope for OCTA. The instrument combines morphological tissue comparison with qualitative dynamic blood flow information to improve the early diagnosis of diseases like bladder cancer (80).

For the time being, OCT is primarily used as a bronchoscopic auxiliary tool to display the microstructure of each layer of the bronchial wall and achieve a similar histopathological diagnosis without tissue biopsy, which helps to reduce the invasive examination and improve the early diagnosis rate of lung cancer, which is critical for the diagnosis and treatment of lung cancer (81). According to Shostak et al., ultra-high resolution images provide essential microstructural information that effectively distinguishes lymph nodes from adjacent airway walls through the characteristics of these microstructures and reveals lymphoid follicles, adipose tissue, pigment-laden histiocytes, and blood vessels information based on needle-based OCT-guided lymph node sampling for lung cancer staging (82).

Hohert et al. used a combination of OCT and autofluorescence imaging (OCT-AFI) to improve diagnostic rates for areas of the lungs not accessible by more extensive imaging methods (83). Furthermore, OCT can aid in determining the tumor’s depth of invasion (84). In malignant lung disease, discriminate between normal and malignant sections of the central airway, lung parenchyma, lymph nodes, and pleura by visualizing illness-related anatomical partitions of the lungs in real-time (85). The results of an *in vitro* scan of 64 specimens of lung nodules suggest that PS-OCT may be able to distinguish between tumors and fibrosis and can be used to guide intraoperative tissue sampling *in vivo* or to assess sufficiency for rapid biopsy *in vitro* (36). Nandy et al. came to a similar conclusion (86).

Presently, the use of OCT in liver cancer is under-reported. In 2015, Zhu et al. performed rapid and high-resolution tomography of human liver specimens using an FF-OCT scanner (87). Nuclear atypia and thicker fibrous bands of hepatocellular carcinoma can be observed on en-face tomography images of FF-OCT. They proposed the support vector machine (SVM) for classifying normal liver tissue and cancerous liver tissue using en-face tomography images. They used the label-free human liver tomography stack to extract seven quantitative parameters, including mean, variance, skewness, kurtosis, energy, entropy, and fractal dimension (FD). The value of FD grows as the liver becomes cancerous, signifying that a divided-dimensional classifier can be utilized for label-free quantitative tumor detection (88).

These encouraging research results suggest that OCT technology will become an important imaging method for deep tumor clinical applications. **Table 3** summarizes the imaging capabilities of OCT in the deep tumor.

**Table 3:** The imaging capabilities of OCT in deep tumors.

Cancer	Authors	Main Findings
Brain cancer	NyúlTóth et al. (2021) (89); Hartmann et al. (2020) (90); Yecies et al. (2019) (10); Tsai et al. (2018) (91)	OCT can not only identify and quantify cerebrovascular morphology and degree of relaxation *in vivo* but also conduct long-term monitoring of cerebrovascular dynamics in dilated. It can also show hidden brain microanatomy to identify brain tumor margins, improving intraoperative safety.
Breast cancer	Mojahed et al. (2020) (74); Kansal et al. (2020) (75); Yang et al. (2020) (76)	FF-OCT has good diagnostic potential in breast surgery and enables real-time assessment of intraoperative margins.
Bladder cancer	Sung et al. (2021) (78); Xu et al. (2021) (79)	OCT can show the depth and type of invasion of urothelial cancer cells, accurately grading and staging bladder cancer. Assist in intraoperative decision-making through real-time disease staging for more accurate diagnosis, resection, and reduced recurrence rates.
Cervical cancer	Chen et al. (2022) (77); Ren et al. (2021) (92); Placzek et al. (2020) (93); Ma et al. (2019) (94); Zeng et al. (2018) (95)	OCT can identify cervical morphological features and lesions noninvasively in real-time.
Lung cancer	Ding et al. (2021) (81)	Endobronchial OCT (EB-OCT) combined with machine learning algorithms can identify malignant lung nodules at a low cost.
Hepatocellular carcinoma	Zhu et al. (2020) (88)	FF-OCT can quantitatively detect hepatocellular carcinoma without markers.

## Conclusions and perspectives

As a mature imaging method being used in new fields, OCT has its unique advantages. Firstly, OCT can provide non-invasive, high-quality detailed images. Through optical, electrical and image processing, OCT can provide micrometer-resolution images of tissues, as well as high-resolution 3D imaging, which can be used for early diagnosis and treatment of diseases. The high soft tissue contrast that OCT can provide facilitates detailed analysis of soft tissue anatomy, which is of great significance for early diagnosis of cancer. In addition, OCT can be integrated into small probes and catheters, making it suitable for entering internal organs for cancer imaging and diagnosis. Due to the limited penetration depth and visual field of OCT itself, it is difficult for an oncologist to diagnose from an image of small area tissue. Besides, the imaging depth is limited in evaluating intraoperative tumor margins, usually within 2mm, which greatly limits its application in surgery. Moreover, it is difficult to fix the probe or imaging module well when imaging with small probes and catheters, so it is difficult to obtain clear images. Therefore, the first step in the future development of OCT is to improve the imaging depth, and combining artificial intelligence algorithms and a variety of imaging methods, so that it can perform imaging at a relatively large depth and field of view. Secondly, under the advantages of ultra-high sampling speed and high resolution of OCT itself, the multi-frame synthesis technology is used to improve the stability of sampling. Finally, OCT will integrate with other disciplines and technologies in the future, such as artificial intelligence, medical image analysis, intelligent machinery manufacturing, safe and environmentally friendly new material processes, et. It can be used not only in early disease diagnosis and facilitating scientific research to provide a more objective and precise imaging measurement basis, but also for the routine detection of diseases to provide safer, faster, and inexpensive technology solutions.

## Author contributions

LY, JM, HZ, and QZ conceived the article. LY and YC wrote the manuscript. SL, JW, GW, and BZ helped to improve the manuscript. All authors have read and agreed to the published version of the manuscript.

## Funding

The work was supported by the Guangdong Basic and Applied Basic Research Foundation (2021A1515011654), Fundamental Research Funds for the Central Universities of China (20720210117), Joint Funds for the Innovation of Science and Technology of Fujian province (2019Y9128), Xiamen Key Laboratory of Endocrine-Related Cancer Precision Medicine (XKLEC2021KF03, XKLEC2020KF05). Key Laboratory of OptoElectronic Science and Technology for Medicine of Ministry of Education, Fujian Provincial Key Laboratory of Photonics Technology (JYG2105). XMU Undergraduate Innovation and Entrepreneurship Training Programs (202210384051, S202210384404, 2021X1119, 2021Y1119, S202110384391), and Shenzhen Bay Laboratory (SZBL2019062801005). Key Laboratory of Nanomedical Technology (Education Department of Fujian Province), School of Pharmacy, Nano Medical Technology Research Institute, Fujian Medical University (2022KLNT201). The Science and Technology Project of Xiamen municipal Bureau of Science and Technology (3502Z20194044).

## Conflict of interest

The authors declare that the research was conducted in the absence of any commercial or financial relationships that could be construed as a potential conflict of interest.

## Publisher’s note

All claims expressed in this article are solely those of the authors and do not necessarily represent those of their affiliated organizations, or those of the publisher, the editors and the reviewers. Any product that may be evaluated in this article, or claim that may be made by its manufacturer, is not guaranteed or endorsed by the publisher.
